# *Prunella vulgaris* L.: An Updated Overview of Botany, Chemical Composition, Extraction Methods, and Biological Activities

**DOI:** 10.3390/ph16081106

**Published:** 2023-08-04

**Authors:** Mussa E. Zholdasbayev, Gayane A. Atazhanova, Safol Musozoda, Ewa Poleszak

**Affiliations:** 1 School of Pharmacy, NJSC “Karaganda Medical University”, Gogol Street, 40, Karaganda 100000, Kazakhstan; g-atazhanova@mail.ru; 2 Department of Pharmaceutical Technology and Pharmacology, Building No. 3, Tajik National University, Rudaki Avenue Street, 17, Dushanbe 734035, Tajikistan; musoev_safol@mail.ru; 3 Department of Applied and Social Pharmacy, Medical University of Lublin, st. Al. Racławickie 1, 20-059 Lublin, Poland; ewa.poleszak@umlub.pl

**Keywords:** *Prunella vulgaris* L., phytochemicals, extracting

## Abstract

*Prunella vulgaris* L. (PV) is a well-known renewable drug resource full of different groups of biologically active substances with a wide range of pharmacological actions and applications in medicine. In this review, we present an updated comprehensive overview of the botany, extracting methods, chemical composition, and pharmacological activity of different parts of PV extracts. As a result of this review, it was found that chemical composition of PV depends on various factors ranging from the part of the plant to the method of extraction. We also highlight extraction methods that have not been previously used for obtaining PV extracts and may have high scientific interest. With this review, we hope to guide present and future professionals and provide possible previously unexplored areas to find new solutions associated with PV plant.

## 1. Introduction

Nowadays, the reduction of pharmaceutical production costs along with increasing the efficacy, quality and safety of medicines is of great interest. The use of domestic types of medicinal plant raw materials seems to be economically viable for many researchers. The *Prunella Vulgaris* L. (PV) has a great scientific interest and practical use as well.

**Figure 1:**
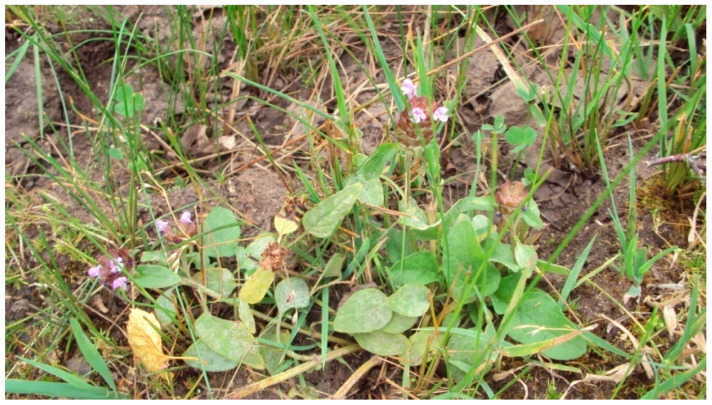
Appearance of *Prunella vulgaris* at the flowering stage.

*Prunella vulgaris* L. (Figure 1) is a perennial herbaceous plant of the *Lamiaceae*/*Labiatae* family, originally in Europe and Asia as a widespread plant of temperate climate. Currently, it has an extensive range and grows in all temperate regions of the world, including Eurasia, Africa, America, and Australia [1].

It usually grows in low mountains and foothills, forest fringe, sparse birch forests and aspen copses, shrubs, wet and sometimes saline meadows and glades, lake shores and oxbow lakes, riverbed gravel stones, and forest roads [2].

*Prunella vulgaris* L. has been successfully used as a drug in European and Chinese traditional medicine since ancient times. *Prunella vulgaris* L. was called a “self-healing” or “all-healing” plant. *Prunella vulgaris* L. fruits are included in the Chinese Pharmacopoeia [3] and the European Pharmacopoeia [4] in 2017. However, the rest of the PV aerial parts are not included in any country’s pharmacopeia, which makes the plant attractive to scientists.

In Europe, *Prunella vulgaris* is approved as a nutritional supplement. It is used as a healing and anti-hemorrhagic agent, as well as to treat sore throats, intestinal infection, and diarrhea. In Asia, the spectrum of PV application is slightly different. It is used as an herbal tea to relieve migraine or fever [5]. In addition, PV has successfully been used to treat thyroid dysfunction and mastitis, as well as pulmonary tuberculosis, infectious hepatitis, and arterial hypertension for more than 2000 years [6]. Goiter, dermatitis, and dermal allergy patients are prescribed PV as well. In addition, it exhibits antioxidant, anti-allergic, anti-inflammatory, and antimicrobial activities [7,8]. Some studies have shown its usefulness in treating atopic dermatitis [9] and photoprotection [10].

Various sources include much information about the properties of the PV aerial part, phytochemical composition, and various types of biological activity due to the popularity of using this plant in medicine. Based on this research, it was found that the main identified components of PV are mono- and sesquiterpenoids, phenolic acids, flavonoids, polysaccharides, pentacyclic triterpenes, higher fatty acids, vitamins, nitrogen-containing compounds, tannins, etc. [11] The caffeic and rosmarinic acids of the phenolic acid class are the basic bioactive agents of PV which determine the anti-inflammatory effect and antioxidant activity [8]. Rosmarinic acid is a complex ester of caffeic and 3-(3,4-dihydroxyphenyl) lactic acid, belonging to the secondary metabolites with a broad spectrum of biological activity. It is one of the effective natural antioxidants [12] which can protect against such free radical pathologies as atherosclerosis, ischaemic heart disease, cancer, and radiation sickness [13]. It is believed that rosmarinic acid binds free radicals of oxygen in the extracellular medium [14,15,16]. Ursolic acid has been described as an effective inhibitor of UVA-modulated expression, the upregulation of which is a visible marker of degradation [17]. It is reported that the amount of rosmarinic acid in the PV aerial mass ranges from 16.8 to 44.4 mg/g [5]. The widespread use of rosmarinic acid is limited by its insufficient production, including the limited raw material base and its manufacturing complexity.

Thus, PV is an important medicinal plant, which, due to its value, rich chemical composition, and pharmacological action, attracts the attention of all scientists in the world. In addition, it is a valuable source of phenolic compounds, flavonoids, and rosmarinic acid, and can serve as a widespread, constantly renewable source of raw materials needed for phytopharmacology. Due to a wide range of PV health benefits, our article provides a comprehensive review of the composition and pharmacological activity, methods for obtaining extracts from PV.

## 2. Botany

*Prunella vulgaris* is a perennial plant 10–50 cm high. It is green and almost glabrous, and its rhizome is creeping and oblique. The stem is erect, simple, and almost glabrous.

The дeaves oblong or ovate, glabrous or sparsely pubescent, entire, sometimes obscurely serrated, 2–6 cm long. The lower leaves on the petioles are longer than the blades, the upper ones are sessile under the inflorescence. The flowers are located in false whorls, in dense capitate, ovoid or oblong terminal, sometimes lateral inflorescences. Bracts broadly ovate or almost round, about 1 cm long, sessile with a heart-shaped base, long pointed, membranous-reticulate, almost glabrous, rarely pubescent, with cilia along the edge, from red to black-violet.

Its calyx is two-lipped, humpbacked above, usually hairy at the base, sessile, or on a short stalk. The upper lip is almost square and flat with three very short sharp teeth.

The corolla purple is 8–12 mm long, 1.5–2 times as long as the calyx. The corolla tube is straight; the upper lip is broadly obovate, concave, and slightly notched at the apex. The lower lip is shorter than the upper ones with an almost round, sharply dentate middle lobe and small rounded ovoid lateral. The filaments of long stamens are located under anther with a subulate, straight or slightly curved process. The nuts are ovoid and elliptical, 1.5–2 mm long, nearly 1 mm wide, trihedral, flat outside, and shiny. The flowering stage occurs between June–September.

It grows on the meadows, forest edges, and glades in shrubs, river valleys and springs, along ditches along the banks of lakes and reservoirs, near housing, and in gardens and orchards. *Prunella vulgaris* is totally spread in Caucasus, Central Asia, Western and Eastern Siberia, the Far East, Western Europe, North Africa, the Balkans, Asia Minor, Iran, India, Tibet, Mongolia, China, and Japan. In Kazakhstan, it is found in the Tobolsk-Ishim, Irtysh, Semipalatinsk, and Kokshetau regions, as well as in Ulutau, Altai, Dzungarian Alatau, and Western Tien Shan [18].

## 3. Extracting Methods of PV

Currently, there are many ways to obtain plant extracts, and each method has its own pros and cons. We reviewed existing methods for obtaining PV extracts and highlighted methods that have not yet previously been used to obtain extracts from PV.

Various extraction methods have been used for PV extraction. Aqueous [19,20,21], methanol [22,23,24,25], ethanol [26,27,28], hexane, and ethyl acetate [29] PV extracts are obtained by traditional methods such as heating, boiling, or refluxing (Table 1). However, the disadvantage of these methods is the loss of flavonoids due to hydrolysis, oxidation, and ionization during extraction, as well as a long extraction time. Currently, there are many alternative modern methods for obtaining extracts from medicinal plants that can solve these problems. Such preparation methods include supercritical [30], accelerated solvent [31], pulsed-electric field [32], enzyme-assisted [33], pressurized liquid extraction [34], ultrasonic [35], and microwave [36] extraction.

Table 1 presents traditional methods of reflux, maceration, and stirring, as well as the efficient extracting methods including ultrasonic, supercritical fluid, and deep eutectic solvent extraction methods which were not previously covered. Ultrasonic extraction is a simple, efficient, and inexpensive alternative to traditional extraction methods. The principle of powerful ultrasound has been associated with the phenomenon of acoustic cavitation, which occurs when high-intensity acoustic waves are generated in a liquid [37]. The extraction mechanism includes two types of physical phenomena: diffusion through the cell walls, and cell content washout after the walls’ destruction [38]. The authors [39] carried out the ultrasonic extraction of PV fruits in order to extract flavonoids in an ultrasonic bath. The extraction parameters are as follows: ethanol extractant from 20 to 60% by volume; liquid/solid ratio from 10:1 to 50:1; the extraction time from 10 to 50 min; and the extraction temperature from 40 to 80 °C. The results showed that the maximum extraction yield of flavonoids by ultrasonic extraction could be 3.62% using 41% (*v*/*v*) ethanol as solvent and a liquid to solid ratio of 30:1 (mL/g) for 30.5 min at 79 °C. In addition, the authors carried out ultrasonic extraction of finely ground PV to evaluate its antiulcer [40] and antitumor [41] properties. Therefore, Xia et al. have presented a new method for PV extraction by using deep eutectic solvents [42]. This method uses natural extraction solvents, and it has such advantages as higher efficiency, economy, and environmental protection, as compared with previously reported conventional extraction methods.

**Table 1:** Methods of extraction of *Prunella vulgaris*.

Method	Plant Part	Solvent	Liquid/Solid Ratio	Temperature, °C	Time	References
Reflux	Aerial part	DI water	-	100	2 h	[19]
Reflux	Herb	DI water	-	70	5 h	[24]
Reflux	Herb	70% ethanol	2:19	-	1 h	[27]
Reflux	Herb	95% ethanol	-	60	4 h	[28]
Stirring	Seed	Methanol	3 × 10 mL methanol g^−1^	-	30 min	[22]
Stirring	Herb	Methanol Hexane Ethyl acetate	9:1	Room temperature	48 h	[29]
Maceration	Leaves, stems, flowers	Methanol DI water	2:1	Room temperature	16 h 24 h	[21]
Maceration	Aerial part	80% methanol	-	Room tempersture	-	[25]
Maceration	Whole plant	Hexane Cloroform Butanol	-	-	-	[26]
Infusing	Leaves of herbal tea	DI water	1:1	80	15 min	[23]
Supercritical fluid extraction	Flowers and dried fruit spikes	-		30	2 h	[30]
Ultrasonic	Leaf, spike inflorescence	DI water 70% methanol	10:1	60	1 h	[20]
Ultrasonic	Herb	from 20 to 60%	from 10:1 to 50:1	From 40 to 80	From 10 to 50	[35]
Deep eutectic solvent extraction	Water-deep eutectic solvent		15 mL·g−^1^	83	42 min	[38]

The extraction using microwave activation is successfully used in world practice to obtain plant extracts [43,44,45]; however, data on PV microwave extraction have not been found in any of the literature. In this regard, the study in this direction becomes promising and may have a high degree of novelty.

## 4. Chemical Composition of PV Plant Parts

The chemical composition of PV is quite diverse and rich in various classes of compounds: mono- and sesquiterpenoids, phenolic acids, flavonoids, polysaccharides, pentacyclic triterpenes, higher fatty acids, vitamins, nitrogen-containing compounds, tannins, etc.

We conducted quantitative analysis and found that PV contains a fairly large amount of flavonoids in the glycosidic form (hyperoside, rutoside), as well as identified smaller amounts of aglycones (quercetol, kaempferol, apigenin) [40]. One of the important flavonoids is hyperoside, which alleviates oxidative damage caused by ROS production [46]. Ursolic and oleanolic acids of the triterpenes in PV are the most common, which have antioxidant, antiallergic, anti-inflammatory and antitumor activities [47]. In addition, Li et al. reported [48] that PV polysaccharides are potential antioxidant and immunomodulatory agents for complementary medicine. *Prunella vulgaris* has been reported to suppress the immune response through immune cell regulation as well [49].

Fazal et al. [50] found that abiotic stresses such as drought, different concentrations of nutrients in the soil, and different light spectra affect the increase in the content of active ingredients, in particular phenolic compounds and flavonoids in PV plants. It has also been defined that the maximum accumulation of the major biologically active substances (BAS) occurs in early flowering. However, high concentrations of BAS are observed from the period of budding through the mass flowering phase. The maximum content of BAS in the aerial part of PV is observed in leaves and inflorescences [51].

We summarized the comparative composition of PV depending on the extracted part of the plant and the selected solvent, described in the literature (Table 2).

Our research topic’s urgency, novelty, and relevance, as well as our 5–10-year literary search, served as the criteria for choosing citation sources in this review. In this regard, new data on chemical constituents from different parts of PV extracts using various solvents, which were not covered earlier in other reviews, have been added to Table 2. The data obtained by the authors of [52,53] showed that rosmarinic acid was the main component of aqueous extracts of PV. To confirm this fact, [52] we additionally carried out the HPLC analysis using a standard sample of rosmarinic acid. It revealed that rosmarinic acid is indeed the main biologically active component of PV.

The authors Isolated two new compounds from the PV seeds extraction, i.e., amolsamic acid A and B, using methanol and found that the main components are rosmarinic and caffeic acids [29]. Another paper [40] published conflicting data on the presence of rosmarinic acid in the methanolic extract of PV seeds.

In the analysis of 50% ethanol extract obtained from the aerial part, five components were identified: chlorogenic, caffeic, and rosmarinic acids, rutin, and quercetin-3-O-glucoside [54]. It was established that the dominant component is rosmarinic acid and the total content of phenolic compounds PV is 65.53 mg/g, whereas the amount of hydroxycinnamic acids is 45.83 mg/g.

Lin et al. found 55 compounds in the PV herb, including 16 flavonoids, 13 phenolic acids, 17 triterpenes, 2 coumarins, and 7 fatty acids [27]. Among them, abscisic acid and daidzein were identified in PV for the first time.

Ozbek et al. obtained methanol, hexane, and ethyl acetate extracts of PV. Two new compounds, i.e., 2-hydroxy-5-methylbenzaldehyde (15.7%) and 5-hydroxymethylfurfural (15.56%), were found in the methanol extract. The hexane extract contained mostly linolenic acid (45.50%), palmitic acid (16.13%), linoleic acid (15.81%), ethyl acetate linoleic acid (14.32%), and palmitic acid (6.21%) [29].

Lin et al. identified 16 components in the PV spike using GC-MS. The basic constituents were squalene (28.03%), linoleic acid (9.96%), and oleic acid (5.51%) [30].

Golembiovska et al. [55] studied the chemical composition of volatile substances of each part of PV (leaves, stems, and roots) grown in Ukraine. They identified 26 hydrocarbons, 13 aldehydes, 10 aromatics, 9 sesquiterpenes, 8 ketones, 7 monoterpenes, 6 acids and esters, and 18 different compounds, where various components predominated in each plant part. According to their study, the main component was squalene, in flowers 164.3 mg/kg, and 156.5 mg/kg in roots.

The result of the total content of phenols in PV seeds is 8.38 ± 0.50 mg/g pyrogallol equivalent (calculated on the dried basis), the share of caffeic acid is 1.85 ± 0.05, and rosmarinic acid is 0.03 ± 0.00% [22]. In addition, according to the data presented by Sárosi et al. [56], the PV seeds are more saturated with phenolic compounds compared to the aerial part.

Tosun et al. studied the effect of various solvents on the total phenolic content (TPC) of PV using the Folin method and defined the order of solvent efficiency: ethyl acetate > acidic acetonitrile > acidic methanol > acidic butanol > acidic water > water > butanol > methanol > acetonitrile > ethyl acetate > acidic hexane > hexane [57]. The TPC of aerial parts of PV aqueous extracts were 90.53 ± 6.81 mg GAE/g [58], and of the whole plant in ethanol, 377.30 ± 12.70 [59].

Mahboubi et al. conducted a study on the content of TPC and total flavonoid content (TFC) of the PV aerial part [60]. The TPC of PV extract was found to be higher in the aqueous extract (156.5 mg GAC/g), while it was (122.1 mg GAC/g) in the ethanol, and (115.7 mg GAC/g) in the methanol ones. The TFC of the PV methanol extract (82.8 mg QE/g) was higher than those of the ethanol extract (22.7 mg QE/g) and the aqueous extract (16.2 mg QE/g). There is an inverse relationship between TPC and TFC based on the choice of solvent. Ahmad G et al. [61] carried out similar studies of the extract obtained from the PV spikes and found that the methanol extract has the highest content of TFC compared to the ethanol and water ones. However, in this study, the evaluation of TPC showed a similar order with TFC, compared to the study mentioned above. This is due to the extraction of different parts of PV which allows us to conclude that the content of TPC and TFC is different.

Therefore, we can conclude that rosmarinic and caffeic acids are the main components and have been found in all parts of PV, which is confirmed by numerous studies in our review. According to the literature data, the chemical composition of PV varies due to the type of solvent, the extractable part of the plant, the method of extracting, and the phase of collection as well. In this regard, there is a great scientific interest in conducting previously unexamined study on the influence of various factors on the chemical composition of PV. According to our viewpoint, there is still no extensive study on the influence of different parameters on the results obtained during extraction of PV, e.g., raw material size, plant part, liquid (solid ratio, solvents), temperature, time, etc. These parameters are important for the chemical composition and following properties of the PV extracts.

## 5. Biological Activities of PV Extracts

Currently, quite a few reviews on the biological properties of PV have been published [62]; however, PV is a plant that still arouses the interest of scientists. Hence, there are new scientific works which have not been covered earlier, but the results of which we would like to examine in this review. For example, there is some new information on the antiviral activity studies (HIV, HSV, Ebola virus, SARS-coronavirus 2) [63,64,65,66].

### 5.1. Antiviral Activity

In a recent review, Mark and Walsh [63] have examined articles reporting potent antiviral activity against HIV and HSV of PV. Another review reports the potential use of aqueous solutions of PV against HIV and Ebola virus [64].

There are literature data on the activity of PV against SARS-coronavirus 2 (SCoV-2) virus infection [65]. The authors demonstrated [66] that an aqueous extract of PV and the compound suramin exhibit a potent inhibitory effect on both wild and mutant (G614) pseudotyped SCoV-2-SP-mediated infections. The IC_50_ for PV and suramin on this type of infection are 30 and 40 µg/mL, respectively. The results of the study showed that the combination of suramin with PV with a neutralizing antibody against SARS-CoV-2 mediated a more powerful blocking effect against SCoV2-SP-PV and therefore can be used as a new antiviral agent against SCoV-2 infection.

Gazanfar et al. conducted a study to assess the hepatoprotective potential of the methanol, aqueous, and ethanol extracts of the PV spike in vivo. Treatment of rats with extracts showed a very significant reduction in serum glutamic oxaloacetic transaminase, serum glutamic pyruvic transaminase, alkaline phosphatase, serum total, and direct bilirubin levels (*p* < 0.01), and a very significant increase in total protein (*p* < 0.01) compared to the toxic control group. This was further supported by histopathological evaluation in which the groups treated with extracts and silymarin had an almost normal liver structure or much less liver damage compared to the group treated with paracetamol. The results of biochemical and histopathological evaluation showed that methanol was the most effective among all the extracts [61].

### 5.2. Antibacterial Activity

Summarized information about the antibacterial activity of PV indicating MIC and MBC with critical attributes is shown in Table 3.

Table 3 presents new references on antibacterial activity [19,29,63]. According to the literature data, the methanol extract of PV has a pronounced antibacterial effect and inhibitory activity against gram-positive bacteria in vitro [8]. Grosan et al. found that methanolic extracts of PV spikes have almost the same antibacterial effect in intensity on both standard strains and clinical isolates (*Pseudomonas aeruginosa*, *Klebsiella pneumoniae*) [67]. In addition to the already published literature, it allows us to conclude that the antibacterial effect of methanol extracts is obtained to a greater extent due to methanol itself, and to a lesser extent, due to the extractable active compounds [68].

Komal et al. showed [19] the growth inhibition of 38 resistant isolates of *Escherichia coli* strains of the PV ethanolic and aqueous extracts in comparison with ciprofloxacin, ofloxacin, cefixime, and tobramycin by the well diffusion method. It has been established that water and alcohol extracts have a positive effect on the multiresistant strain of *Escherichia coli*, separately and in combination with each other. In this regard, there is likelihood of using PVs as a maintenance therapy in parallel with standard antibiotics used to treat urinary tract infections.

Patel et al. [69], in their study, tested the in vitro methanol and petroleum ether extracts of PV for activity against *Bacillus subtilis*, *Escherichia coli*, *Staphylococcus aureus*, and *Salmonella typhi*. Similarly, the in vivo studies were conducted by using peritonitis caused by *Escherichia coli* on laboratory rats where they were given ofloxacin allopathic antibiotics. The results were compared with those rats which were given herbal extracts under controlled conditions. The study has defined that the petroleum ether extracts exhibit the lowest antibacterial activity compared to the methanol extracts. The PV methanolic extracts effectively restrict the growth of *Escherichia coli* at 100 µg/mL, *Bacillus subtilis* at 50 µg/mL, and *Staphylococcus aureus* at 100 µg/mL, while *Salmonella typhi* showed complete resistance.

In a recent study, [29] the authors have evaluated the antibacterial activity of methanol, ethyl acetate, and hexane extracts of PV against *Bacillus cereus*, *Clostridium perfringens*, *Staphylococcus aureus*, *Streptococcus pyogenes*, *Salmonella typhimurium*, *Escherichia coli*, *Serratia marcescens*, *Klebsiella pneumoniae*, *Proteus vulgaris*, and *Pseudomonas aeruginosa*. The methanol extract showed the highest antibacterial activity against *Bacillus cereus*, hexane against *Salmonella typhimurium*, and ethyl acetate against *Streptococcus pyogenes*. We can conclude that these extracts can be used in the future as antibacterial agents to prevent diseases associated with these microbial infections.

### 5.3. Antitumor Activity

The cell count kit-8 (CCK-8) on cancer cells from the SCC154 oral squamous cell carcinoma cell line was used in order to assess the antitumor effect of PV distillate. The PV distillate was found to be cytotoxic for SSC154 cancer cells depending on the dosage [41].

According to the study [70], the main components that determine the antitumor properties of PV are caffeic and rosmarinic acids. In this study, chemotherapy was performed in combination with a taxane for treating patients with breast cancer. The mean follow-up time was 41 months. The PV treatment improved a complete response rate and an overall survival time compared to those in the control group (*p* < 0.05). The three-year overall survival rates were 86.5 and 77.2% among patients in the experimental and control groups, respectively (*p* < 0.05). In addition, the PV treatment prevents side effects, namely neutrophil fever and chemotherapy-induced anemia. Therefore, PV may be a potential adjuvant in treating breast cancer.

Our study showed that the extract (concentrated aqueous solution after initial methanol extraction) from the PV spikes had dose- and time-dependent cytotoxic and anti-migratory potential in humans on a tested breast adenocarcinoma cell line, namely MDA-MB-231, and at the same time, affected non-tumor epithelial cells of the mammary gland to a lesser extent [67].

Antitumor activity was also tested by Hwang et al. using HepG2, HT29, A549, MKN45, and HeLa cancer cell lines. The PV ethanol extract (at 50 and 100 µg/mL), hexane fraction (at 100 µg/mL), and chloroform fraction (at 50 and 100 µg/mL) inhibited cell proliferation, but not at 10 µg/mL. Conversely, in groups receiving 10 µg/mL of ethanol extract or butanol fraction or 10, 50, and 100 µg/mL of aqueous fraction, the proliferative effect exceeded 10%. The results showed that the PV ethanol extract caused significant cytotoxic effects on various cancer cell lines [26].

Feng et al. found that a 60% ethanol extract of PV inhibited C57BL/6 mice’s tumor growth, increased superoxide dismutase activity, and reduced serum malondialdehyde levels in tumor-bearing mice. These results indicate that a 60% ethanol extract of PV has a high antioxidant activity both in vitro and in vivo. This extract may play an important role in the prevention and treatment of tumors and be useful as a dietary supplement or an antitumor agent [71].

In vitro, the PV extract has activity against human uterine myoma (UM) cells. It does not show drug toxicity and promotes apoptosis of human UM cells, as well as inhibits the transition of UM cells from the G0/G1 stage to the G2 stage, where the DNA replication occurs. In vivo, PV has an anti-UM activity, reduces estrogen and progesterone concentrations, downregulates estrogen and progesterone receptor expression levels via the estrogen signaling pathway, and promotes UM cell apoptosis by decreasing survivin and Bcl-2 protein expression levels and increasing caspase-3 and Bax expression levels through the mitochondria-mediated pathway of apoptosis [30].

### 5.4. Antioxidant Activity

Total flavonoid (TFC) content including rosmarinic acid, caffeic acid, and hyperoside directly affects the antioxidant capacity (DPPH• and ABTS•+). Chen et al. [72] revealed a pattern that during the growth of spika, TFC significantly decreased, while the content of salviaflazid increased. Spika had the highest TFC and rosmarinic and caffeic acids, as well as the highest antioxidant activity at the flowering stage, and the highest hyperoside content in a bud-formation period.

An experiment described by Seo et al. [73] with a methanolic extract of PV flowering stems showed a DPPH inhibitory activity of 35.4% and an ABTS inhibitory activity of 61.7% at a concentration of 100 µg/mL. Hwang et al. [26] found that the methanol extract of PV seeds is much more active than the extract obtained from the PV flowering stems.

In this study, the results of the assessment of the PV oxidant effect showed that the ethanol and aqueous extract of PV have a higher content of antioxidants compared to other solvent fractions, similar to their total content of phenols [28]. A 60% ethanol extract of PV showed high activity in removing free radicals in vitro according to the ABTS, DPPH, and FRAP methods [73]. Total phenols strongly correlated with antioxidant activity (R2 = 0.9988 in ABTS•+; 0.6284 in DPPH, and 0.9673 in FRAP tests).

Other authors have obtained different results and found that the organic fraction has a more pronounced antioxidant activity than the aqueous ones. Psotova et al. [8] isolated rosmarinic acid (25.7% *w*/*w*) and caffeic acid (0.37%) from the aerial part of PV by the methanol extraction followed by the organic fraction isolation. The IC_50_ values for rosmarinic and caffeic acids were 1.87 ± 0.06 and 0.81 ± 0.05 mg/mL, respectively.

The study obtained data on a high-antioxidant, antiradical, and low hemolytic activity of 50% PV ethanol extract. Low hemolytic activity is observed in aqueous 25%, as well as in 50% PV leaf extracts. Both 50% and 96% ethanol extracts have high antioxidant activities [74].

### 5.5. Treatment of Thyroiditis-Associated Diseases

Traditional Chinese medicine has been treating thyroid diseases with PV for thousands of years. There are many studies on a potential therapeutic effect of the PV extracts on autoimmune thyroiditis and other types of thyroiditis-dependent diseases [75]. Serum thyroglobulin antibodies (TgAb) and thyroid volume in rats in an experimental model of autoimmune thyroiditis (EAT) dropped sharply after administrating an aqueous extract of PV spikes. In addition, PV induced indoleamine-2,3 dioxygenase (IDO1) mRNA and protein expression in the spleen and intestines of rat EAT treated with PV [52].

Han et al. conducted 13 randomized clinical trials involving 1468 patients. A meta-analysis has shown that PV in combination with levothyroxine sodium tablets or thyroxine tablets has more benefits in thyroid nodules, further increasing clinical efficacy, reducing the diameter of nodules, and reducing the frequency of adverse reactions [76].

The molecular docking studies have shown that luteolin and kaempferol are BAS of PV which may play an important role in treating Hashimoto’s thyroiditis by regulating several signaling pathways: TNF, MAPK (mitogen-activated protein kinase), and PI3K-Akt [77]. As a result of the KEGG pathway network and target compound analysis, it was found that quercetin, luteolin, kaempferol, and beta-sitosterol are the main BAS in PV, responsible for activity in treating subacute thyroiditis, and are closely associated with their regulation of inflammation and apoptosis by targeting PIK3CG proteins, MAPK1, MAPK14, TNF and PTGS2, and PI3K-Akt and TNF signaling pathways. The study has shown that quercetin, luteolin, kaempferol, and PV beta-sitosterol may play key roles in the treatment of subacute thyroiditis associated with the regulation of inflammation and apoptosis by influencing PI3K-Akt and TNF signaling pathways [78].

The effect of PV polysaccharides on cultured orbital fibroblasts in vitro among the thyroid-associated ophthalmopathy (TAO) patients has been studied as well. *Prunella vulgaris* polysaccharides have been used in different concentrations to treat different groups of fibroblasts among both TAO patients and healthy ones. Dexamethasone was used as a positive control drug, whereas interferon-γ (IFN-γ) was used as a positive stimulator. The results showed that the PV polysaccharides at concentration above 400 µg/mL significantly inhibited orbital fibroblast proliferation among the TAO patients (*p* < 0.05). However, no definite inhibitory effect was found in orbital fibroblasts of healthy patients. *Prunella vulgaris* polysaccharides in combination with dexamethasone in all concentrations can significantly promote apoptosis of orbital fibroblasts when co-cultured with IFN-γ (*p* < 0.05). Our study results suggest that PV has a therapeutic effect by inhibiting the orbital fibroblast proliferation and promoting apoptosis of orbital fibroblasts among the TAO patients [79].

### 5.6. Other Activities

Grosan et al. have identified that the hydroalcoholic extracts of PV in an in vivo experiment have a dose-dependent antiulcer effect due to a rich content of polyphenols which have a protective effect for stomach ulcers when taking the non-steroidal anti-inflammatory drugs (NSAIDs) [40].

The aqueous and methanolic extracts of PV were evaluated for anthelmintic efficacy against ovine gastrointestinal nematodes both in vitro and in vivo using the worm motility inhibition assay, egg hatch assay, and faecal egg reduction percentage assay, respectively. The methanol extract (LC_50_ = 2.48 mg/mL) has a stronger inhibitory effect compared to an egg hatching aqueous extract (LC_50_ = 3.36 mg/mL) showing a higher ovicidal activity. In vivo the largest reduction of fecal eggs (92.86%) was recorded for a crude methanol extract, followed by a crude aqueous extract (80.34%) at 2 g/kg body weight on the 15th post-treatment day [21]. The present study’s results suggest that the PV extracts are promising alternatives to commercially available anthelmintic agents.

The methanolic PV seed extract showed a strong anti-inflammatory activity on neutrophils, myeloperoxidase, and horseradish peroxidase models. The anti-inflammatory effect of PV seed extract was also characterized by its ability to modulate the myeloperoxidase enzyme activity [22]. The results of the study showed that treatment with PV tea (water extract of leaves) significantly increased inducible nitric oxide synthase and demonstrated anti-inflammatory effects through the 5-lipoxygenase, myeloperoxidase, and nitric oxide synthase pathways [23].

Anti-IHNV (infectious hematopoietic necrosis virus) activity was studied on 32 medicinal plants using cyprini papulosum epithelioma (EPC) cells. Among these plants, PV showed the strongest inhibition of IHNV replication, with a 99.3% inhibition rate at 100 mg/L. In addition, it was found that the IC_50_ of ursolic acid after 72 h on IHNV is 8.0 μM. In addition, UA can significantly reduce a cytopathic effect and virus titer induced by IHNV in EPC cells [80].

An aqueous methanolic extract of the PV aerial part increased wound strength by 33.9% in a linear wound model and caused a 75.2% contraction on the 12th day in a circular wound model. In a postoperative wound model, the ethyl acetate extract increased wound tensile strength by 39.3%, whereas the 86.3% reduction was found in the excisional wound model on the 12th day [25].

All PV parts have antihypertensive effects in the clinical management of hypertension. An aqueous extract and a water-ethanol (30%) extract of PV reduce animals’ blood pressure [81].

In a study conducted by Lin et al., the results showed that the PV fraction eluted with 70% ethanol had a significant hypnotic effect that shortened sodium pentobarbital-induced mice’s sleep latency (*p* < 0.01) and prolonged their sleep time (*p* < 0.01), compared to the control group. In addition, the fraction eluted with water, as well as 20% ethanol, and 95% ethanol (*p* > 0.05), had no significant differences in comparison with the control group and had no hypnotic effect [27].

## 6. Dosage Forms and Clinical Applications

In recent years, the PV-based drugs have been of great interest in clinical practice, used both as a separate substance and in combination with other drugs and non-drugs. Currently, PV is used to produce various drugs such as injections, oral liquid, and ointment. As previously reported in this review, drug efficiency will depend on the PV extraction methods, the type of solvent used, and the technological process as well.

According to the Order of the Ministry of Health of the Republic of Belarus, dated February 25, 1998, No. 56, PV is included in the Nomenclature of Homeopathic Medicines. PV is an active ointment substance for treating burns [82]. It is included in the Liren Bio-lipopolis anti-cellulite firming gel (Lab. Dr Irena Eris Production), a mixture of plant raw materials used to produce the mammoleptin capsules for treating fibrocystic mammopathy, as well as the Frudia face mask (WelcosCo. LTD Production, South Korea). PV is actively used by manufacturers to produce dietary supplements called Selfheal in the form of tablets, and liquid extracts based on PV (manufacturers: Nature’s Health, Secrets of the Tribe, HawaiiPharm).

In order to be used in the form of tablets, the PV extract is dried and compressed. The drug in this form has a broad spectrum of activity, including good results in the treatment of benign prostatic hyperplasia [83]. The authors conducted taxane chemotherapy together with the oral form of PV, which found high efficacy and safety in treating breast cancer patients [70]. In addition, we have defined that the PV tablets in combination with glucocorticoids have a shorter treatment course and fewer adverse reactions in the treatment of acute thyroiditis [84]. According to a study of thyroiditis by Fan et al., the PV-based capsules in combination with levothyroxine sodium tablets significantly increased thyroid levels, compared to only l-thyroxine-treated patients [85].

## 7. Conclusions

Relevant PV studies are globally carried out from year to year. Thus, this article summarizes the study status on phytochemistry, vegetation regions, and methods for obtaining pharmacological effects over the last decades. With this review, we hope to guide future professionals in natural plant research and provide possible previously unexplored areas to find new solutions.

First, PV has a rich chemical composition, including triterpenoids and their glycosides, flavonoids, phenolic acids and their glycosides, organic acids, sterols, essential oils, polysaccharides, and tannins. However, further studies are needed to understand the plant’s future application, as well as to identify the metabolites responsible for a particular type of activity. According to current global trends, BAS should be extracted and purified using advanced economical and green technologies. For instance, no literature data were found concerning the use of microwave activation to obtain PV extracts. This method is environmentally friendly in terms of reducing the extraction time by 5–20 times compared to traditional ones. The use of microwave extraction has a high scientific potential due to releasing and obtaining new substances, and the quantitative content of BAS from plant raw materials, in particular from PV, which may cause other properties different from the already available data.

Secondly, despite its rich chemical composition and high biological activity, PV is not so widely used in medicine, which causes scientific interest in the development of dosage forms based on PV that have not yet been developed (e.g., an ointment, a gel, a cream, a syrup, powders, suppositories, suspensions, etc.).

Thus, we conclude that PV is a powerful and widespread drug resource with a huge potential for development. Therefore, there is a need to additionally study all aspects of the isolation, preparation, and identification of BAS, extraction methods, solvent selection, and operating conditions for obtaining dosage forms for maximum release, as well as determining quality standards and ensuring product safety.

## Figures and Tables

**Table 2:** Chemical compositions of different plant parts of *Prunella vulgaris*.

№	Compound Name	RT	Method	Solvent	Plant Part	Reference
1	Gluconic acid 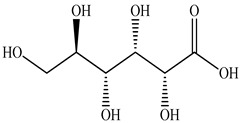	0.88	UPLC-ESI-MS	Water	Spike	[47]
2	Malic acid 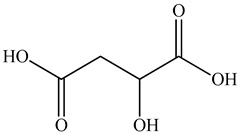	1.05	UPLC-ESI-MS	Water	Spike	[47]
3	Citric acid 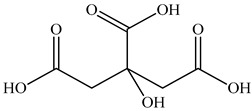	1.26	UPLC-ESI-MS	Water	Spike	[47]
		6.58	UPLC-MS/MS	Ethanol/Water	Whole plant	[27]
4	Caftaric acid 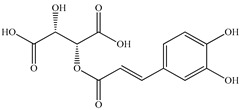	2.10	HPLC-UV/MS	Methanol/Water	Seed	[36]
5	Gentisic acid 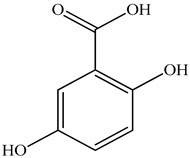	2.15	HPLC-UV/MS	Methanol/Water	Seed	[36]
6	Danshensu 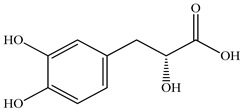	2.62	UPLC-MS/MS	Ethanol/Water	Whole plant	[27]
7	Long-chain fatty acid 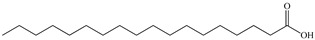	2.65	UPLC-ESI-MS	Water	Spike	[47]
8	Protocatechuic acid 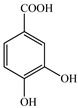	3.25	UPLC-ESI-MS	Water	Spike	[47]
		5.79	HPLC-ESI-MS/M	Water	Spike	[48]
9	m-Dimethylbenzene 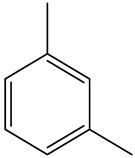	4.76	GC-MS	Ethyl acetate	Spike	[30]
10	Protocatechuic aldehyde 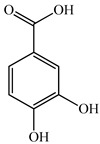	5.85	UPLC-ESI-MS	Water	Spike	[47]
		7.06	HPLC-ESI-MS/M	Water	Spike	[48]
11	Chlorogenic acid 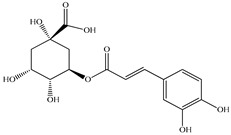	5.01	UPLC-MS/MS	Ethanol/Water	Whole plant	[27]
		5.6	HPLC-UV/MS	Methanol/Water	Seed	[36]
		13.01	HPLC	Ethanol/Water	Aerial part	[49]
12	Thymine 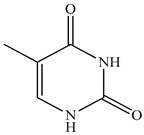	5.39	GC-MS	Methanol	Whole plant	[29]
13	Dihydrocaffeic acid 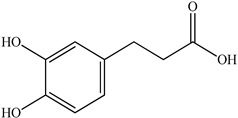	5.65	UPLC-MS/MS	Ethanol/Water	Whole plant	[27]
14	Caffeic acid 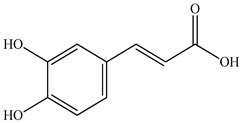	5.6	HPLC-UV/MS	Methanol/Water	Seed	[36]
		6.67	UPLC-MS/MS	Ethanol/Water	Whole plant	[27]
		8.46	HPLC-ESI-MS/M	Water	Spike	[48]
		9.17	UPLC-ESI-MS	Water	Spike	[47]
		14.08	HPLC	Ethanol/Water	Aerial part	[49]
		20.99	HPLC	Methanol	Seed	[22]
15	Esculetin 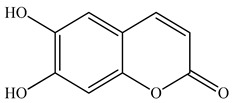	5.85	UPLC-MS/MS	Ethanol/Water	Whole plant	[27]
16	Trans-caran 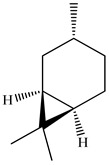	6.13	GC-MS	Hexane	Whole plant	[29]
17	2-dodecan 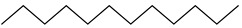	6.2	GC-MS	Ethyl acetate	Whole plant	[29]
18	Coumaran 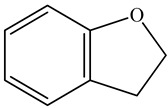	6.43	GC-MS	Methanol	Whole plant	[29]
19	5-hydroxymethylfurfural 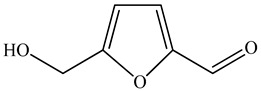	6.53	GC-MS	Methanol	Whole plant	[29]
20	Carvacrol 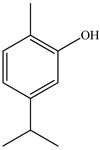	7.05	GC-MS	Hexane	Whole plant	[29]
		7.14	GC-MS	Ethyl acetate	Whole plant	[29]
21	Thymol 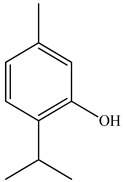	7.05	GC-MS	Ethyl acetate	Whole plant	[29]
		7.14	GC-MS	Hexane	Whole plant	[29]
22	Eugenol methylether 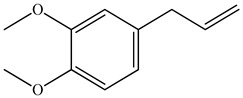	8.25	GC-MS	Methanol	Whole plant	[29]
23	P-Coumaric acid 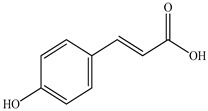	8.7	HPLC-UV/MS	Methanol/Water	Seed	[27]
24	2-hydroxy-5-methylbenzaldehyde 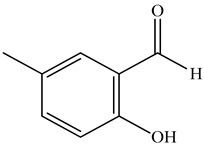	8.7	GC-MS	Methanol	Whole plant	[29]
25	D-Limonene 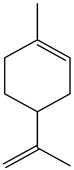	8.5	GC-MS	Water	Roots	[50]
		9.61	GC-MS	Ethyl acetate	Spike	[30]
26	Mangiferin 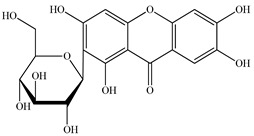	9.96	UPLC-MS/MS	Ethanol/Water	Whole plant	[27]
27	Isorosmarinic acid glycoside 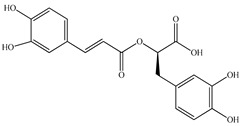	10.05	UPLC-ESI-MS	Water	Spike	[47]
28	Scopoletin 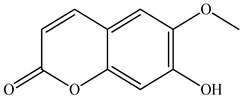	10.78	UPLC-MS/MS	Ethanol/Water	Whole plant	[27]
29	Rosmarinic acid 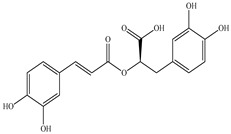	10.88	UPLC-ESI-MS	Water	Spike	[47]
		12.08	HPLC-ESI-MS/M	Water	Spike	[48]
		20.51	HPLC	Ethanol/Water	Aerial part	[49]
		21.09	UPLC-MS/MS	Ethanol/Water	Whole plant	[27]
		33.1	HPLC	Methanol	Seed	[22]
30	Linalool 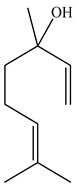	11.48	GC-MS	Ethyl acetate	Roots	[50]
31	Ferulic acid 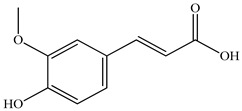	12.10	UPLC-MS/MS	Ethanol/Water	Whole plant	[27]
		12.2	HPLC-UV/MS	Methanol/Water	Seed	[36]
32	Ursolic acid 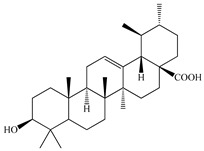	12.10 47.21	UPLC-ESI-MS UPLC-ESI-MS	Water Ethanol/Water	Spike Whole plant	[47] [27]
33	Isoorientin 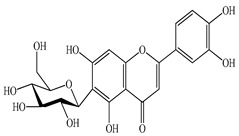	12.47	UPLC-MS/MS	Ethanol/Water	Whole plant	[27]
34	Dihydroferulic acid 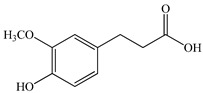	14.05	UPLC-MS/MS	Ethanol/Water	Whole plant	[27]
35	Caprylic acid 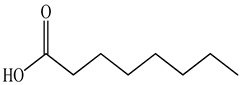	14.13	GC-MS	Ethyl acetate	Leaves	[50]
36	Jacoesidin 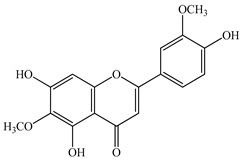	14.58	UPLC-MS/MS	Ethanol/Water	Whole plant	[27]
37	Cynaroside 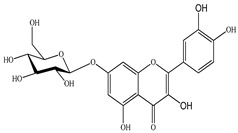	15.88	UPLC-MS/MS	Ethanol/Water	Whole plant	[27]
38	Hyperoside 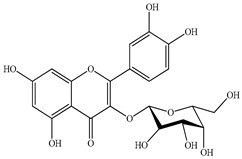	16.26	UPLC-MS/MS	Ethanol/Water	Whole plant	[27]
		18.6	HPLC-UV/MS	Methanol/Water	Seed	[36]
39	Rutin 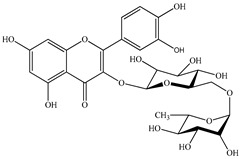	16.92	UPLC-MS/MS	Ethanol/Water	Whole plant	[27]
		19.76	HPLC	Ethanol/Water	Aerial part	[49]
		20.2	HPLC-UV/MS	Methanol/Water	Seed	[36]
40	Lavandulol acetate 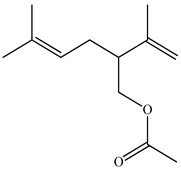	17.26	GC-MS	Ethyl acetate	Whole plant	[29]
41	β-Cyclocitrale 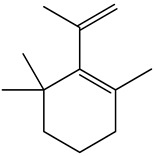	17.31	GC-MS	Ethyl acetate	Leaves	[50]
42	Salviaflaside 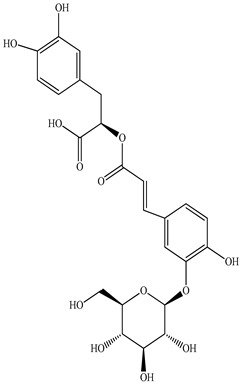	17.76	UPLC-MS/MS	Ethanol/Water	Whole plant	[27]
		29.2	HPLC	Methanol	Seed	[22]
43	Genistein 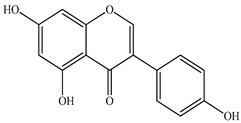	18.5	UPLC-MS/MS	Ethanol/Water	Whole plant	[27]
44	Hesperidin 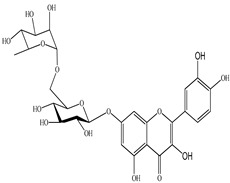	19.08	UPLC-MS/MS	Ethanol/Water	Whole plant	[27]
45	Isoquercitrin 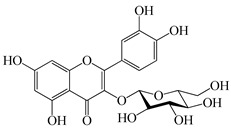	19.6	HPLC-UV/MS	Methanol/Water	Seed	[36]
46	Abscisic acid 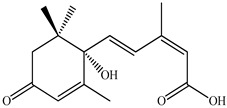	19.19	UPLC-MS/MS	Ethanol/Water	Whole plant	[27]
47	Kaempferol-7-O-glucoside 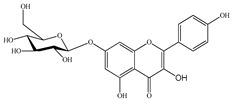	19.56	UPLC-MS/MS	Ethanol/Water	Whole plant	[27]
48	β-Bourbonene 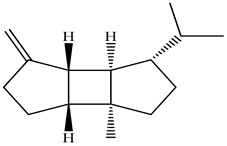	19.98	GC-MS	Ethyl acetate	Leaves	[50]
49	Quercitin 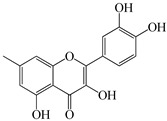	20.24	HPLC	Ethanol/Water	Aerial part	[49]
		23.0	HPLC-UV/MS	Methanol/Water	Seed	[36]
50	Methyl caffeate 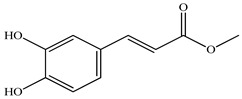	21.22	UPLC-MS/MS	Ethanol/Water	Whole plant	[27]
51	β-Farnesene 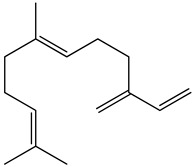	21.56	GC-MS	Ethyl acetate	Roots	[50]
52	Humulene 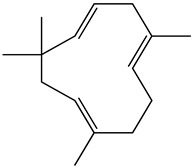	22.01	GC-MS	Ethyl acetate	Roots	[50]
53	γ-Nonalactone (Prunolide) 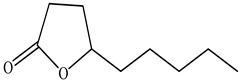	22.31	GC-MS	Ethyl acetate	Stems	[50]
54	Geranyl acetone 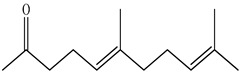	22.53	GC-MS	Ethyl acetate	Stems	[50]
55	Germacren D 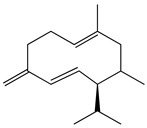	22.66	GC-MS	Ethyl acetate	Leaves	[50]
56	Cyanidin 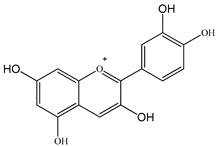	24.97	UPLC-MS/MS	Ethanol/Water	Whole plant	[27]
57	Phytol 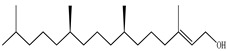	25.13	GC-MS	Ethyl acetate	Whole plant	[29]
		25.15	GC-MS	Methanol	Whole plant	[29]
58	Spathulenol 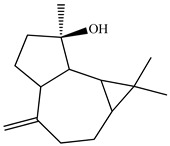	25.23	GC-MS	Ethyl acetate	Flowers, leaves	[50]
59	Ethyl caffeate 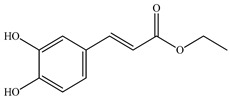	25.34	UPLC-MS/MS	Ethanol/Water	Whole plant	[27]
60	Quercetol 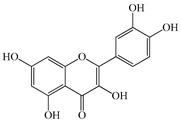	26.8	HPLC-UV/MS	Methanol/Water	Seed	[36]
61	Myristic acid 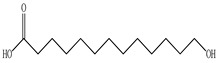	27.15	GC-MS	Ethyl acetate	Leaves, stems, roots	[50]
62	Acacetin 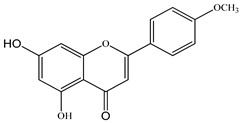	27.81	UPLC-MS/MS	Ethanol/Water	Whole plant	[27]
63	Methyl rosinate 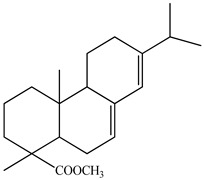	28.44	UPLC-MS/MS	Ethanol/Water	Whole plant	[27]
64	Pentadecane 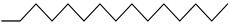	28.80	GC-MS	Ethyl acetate	Spike	[30]
65	Luteolin 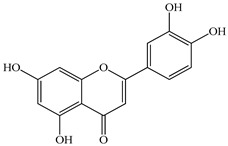	29.64	UPLC-MS/MS	Ethanol/Water	Whole plant	[27]
66	Kaempferol 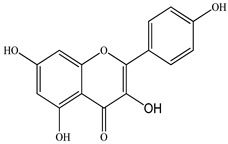	31.6	HPLC-UV/MS	Methanol/Water	Seed	[36]
		32.79	UPLC-MS/MS	Ethanol/Water	Whole plant	[27]
67	Ethyl rosemary 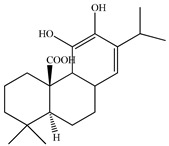	32.38	UPLC-MS/MS	Ethanol/Water	Whole plant	[27]
68	Hexadecane 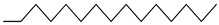	23.18	GC-MS	Ethyl acetate	Flowers, leaves, stems, roots	[50]
		32.53	GC-MS	Ethyl acetate	Spike	[30]
69	Apigenin 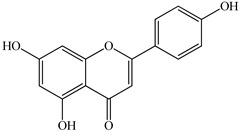	33.1	HPLC-UV/MS	Methanol/Water	Seed	[36]
		33.51	UPLC-MS/MS	Ethanol/Water	Whole plant	[27]
70	Daidzein 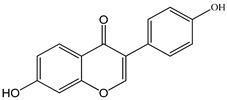	35.93	UPLC-MS/MS	Ethanol/Water	Whole plant	[36]
71	Heptadecane 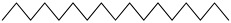	24.84	GC-MS	Ethyl acetate	Flowers, leaves, stems, roots, spikes	[50]
		32.53	GC-MS	Ethyl acetate		[30]
72	Amolsamic acid A 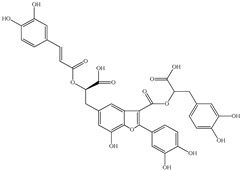	40.2	HPLC	Methanol	Seed	[22]
73	Amolsamic acid B 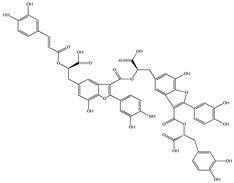	41.6	HPLC	Methanol	Seed	[22]
74	Prunelloside A 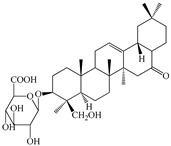	46.09	UPLC-MS/MS	Ethanol/Water	Whole plant	[27]
75	Maslinic acid 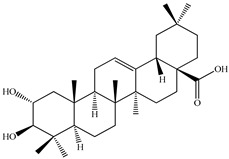	46.33	UPLC-MS/MS	Ethanol/Water	Whole plant	[27]
76	Corosolic acid 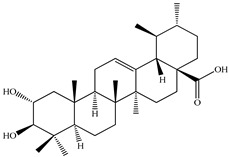	46.74	UPLC-MS/MS	Ethanol/Water	Whole plant	[27]
77	Oleanic acid 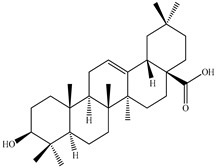	46.95	UPLC-MS/MS	Ethanol/Water	Whole plant	[27]
78	Linolenic acid 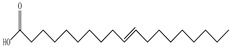	25.90	GC-MS	Methanol	Whole plant	[29]
		25.92	GC-MS	Ethyl acetate	Whole plant	[29]
		25.99	GC-MS	Hexane	Whole plant	[29]
		47.02	UPLC-MS/MS	Ethanol/Water	Whole plant	[27]
79	Linoleic acid 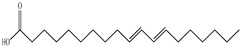	25.70	GC-MS	Methanol	Whole plant	[29]
		25.72	GC-MS	Ethyl acetate	Whole plant	[29]
		25.79	GC-MS	Hexane	Whole plant	[29]
		47.45	UPLC-MS/MS	Ethanol/Water	Whole plant	[27]
		51.18	GC-MS	Ethyl acetate	Spike	[30]
80	Palmitic acid 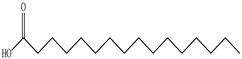	20.73	GC-M	Methanol	Whole plant	[29]
		20.74	GC-MS	Ethyl acetate	Whole plant	[29]
		20.8	GC-MS	Hexane	Whole plant	[29]
		29.84	GC-MS	Ethyl acetate	Leaves	[50]
		45.67	GC-MS	Ethyl acetate	Spike	[30]
		47.90	UPLC-MS/MS	Ethanol/Water	Whole plant	[27]
81	Oleic acid 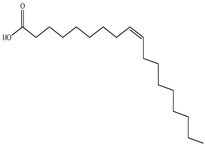	32.15	GC-MS	Ethyl acetate	Leaves, roots	[50]
		48.01	UPLC-MS/MS	Ethanol/Water	Whole plant	[27]
82	Stearic acid 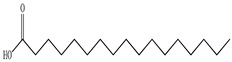	26.52	GC-MS	Ethyl acetate	Whole plant	[29]
		26.55	GC-MS	Methanol	Whole plant	[29]
		48.82	UPLC-MS/MS	Ethanol/Water	Whole plant	[27]
83	Squalene 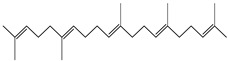	38.7	GC-MS	Ethyl acetate	Flowers, roots	[50]
		67.36	GC-MS	Ethyl acetate	Whole plant	[29]

**Table 3:** Antibacterial activity of *Prunella vulgaris* extracts.

Type of Extract	Plant Part	Strain	MIC	MBC	Reference
Organic Fraction of PV	Herb	*S. aureus* ATCC3953	256 mg/mL	-	[8]
		*S. aureus* ATCC4223	128 mg/mL	-	
Methanolic	Leaves	*K. pneumoniae* ATCC13883	25 mg/mL	50 mg/mL	[63]
		*E. coli* ATCC25922	50 mg/mL	50 mg/mL	
		*P. aeruginosa* ATCC27853	12.5 mg/mL	50 mg/mL	
		MRSA ATCC 43300	25 mg/mL	25 mg/mL	
	Flowers	*K. pneumoniae* ATCC13883	25 mg/mL	25 mg/mL	[63]
		*E. coli* ATCC25922	25 mg/mL	25 mg/mL	
		*P. aeruginosa* ATCC27853	6.25 mg/mL	25 mg/mL	
		MRSA ATCC 43300	25 mg/mL	25 mg/mL	
Aqueous	Leaves	*K. pneumoniae* ATCC13883	>50 mg/mL	>50 mg/mL	[63]
		*E. coli* ATCC25922	>50 mg/mL	>50 mg/mL	
		*P. aeruginosa* ATCC27853	>50 mg/mL	>50 mg/mL	
		MRSA ATCC 43300	>50 mg/mL	>50 mg/mL	
	Flowers	*K. pneumoniae* ATCC13883	50 mg/mL	>50 mg/mL	[63]
		*E. coli* ATCC25922	>50 mg/mL	>50 mg/mL	
		*P. aeruginosa* ATCC27853	25 mg/mL	>50 mg/mL	
		MRSA ATCC 43300	>50 mg/mL	>50 mg/mL	
Aqueous	Whole plant	*E. coli* ATCC25922	2 μg/mL	-	[19]
Ethanolic	Whole plant	*E.coli* ATCC25922	1.33 μg/mL	-	[19]
Ethyl acetate	Whole plant	*C. perfringens*	1.56 μg/mL	-	[29]
		*S. aureus*	6.25 μg/mL	-	
		*E. coli*	0.195 μg/mL	-	
		*S. marcescens*	6.25 μg/mL	-	
		*K. pneumoniae*	0.78 μg/mL	-	
		*P. aeruginosa*	1.56 μg/mL	-	
Hexane	Whole plant	*C. perfringens*	6.25 μg/mL	-	[29]
		*S. pyogenes*	3.12 μg/mL	-	
		*S. typhimurium*	1.56 μg/mL	-	
		*E. coli*	6.25 μg/mL	-	
		*P. vulgaris*	3.12 μg/mL	-	
		*P. aeruginosa*	6.25 μg/mL	-	
Methanolic	Whole plant	*B. cereus*	1.56 μg/mL	-	[29]
		*C. perfringens*	6.25 μg/mL	-	
		*S. marcescens*	6.25 μg/mL	-	

## Data Availability

Not applicable.
